# Patient Portals Facilitating Engagement With Inpatient Electronic Medical Records: A Systematic Review

**DOI:** 10.2196/12779

**Published:** 2019-04-11

**Authors:** Ronald Dendere, Christine Slade, Andrew Burton-Jones, Clair Sullivan, Andrew Staib, Monika Janda

**Affiliations:** 1 Centre for Health Services Research Faculty of Medicine The University of Queensland Woolloongabba Australia; 2 Institute for Teaching and Learning Innovation The University of Queensland Brisbane Australia; 3 School of Business Faculty of Business, Economics and Law The University of Queensland Brisbane Australia; 4 Metro North Hospital and Health Service Department of Health Queensland Government Brisbane Australia; 5 Metro South Hospital and Health Service Department of Health Queensland Government Brisbane Australia; 6 University of Queensland School of Medicine Brisbane Australia

**Keywords:** patient portal, electronic medical record, electronic health record, patient engagement, digital hospital

## Abstract

**Background:**

Engaging patients in the delivery of health care has the potential to improve health outcomes and patient satisfaction. Patient portals may enhance patient engagement by enabling patients to access their electronic medical records (EMRs) and facilitating secure patient-provider communication.

**Objective:**

The aim of this study was to review literature describing patient portals tethered to an EMR in inpatient settings, their role in patient engagement, and their impact on health care delivery in order to identify factors and best practices for successful implementation of this technology and areas that require further research.

**Methods:**

A systematic search for articles in the PubMed, CINAHL, and Embase databases was conducted using keywords associated with patient engagement, electronic health records, and patient portals and their respective subject headings in each database. Articles for inclusion were evaluated for quality using A Measurement Tool to Assess Systematic Reviews (AMSTAR) for systematic review articles and the Quality Assessment Tool for Studies with Diverse Designs for empirical studies. Included studies were categorized by their focus on input factors (eg, portal design), process factors (eg, portal use), and output factors (eg, benefits) and by the valence of their findings regarding patient portals (ie, positive, negative, or mixed).

**Results:**

The systematic search identified 58 articles for inclusion. The inputs category was addressed by 40 articles, while the processes and outputs categories were addressed by 36 and 46 articles, respectively: 47 articles addressed multiple themes across the three categories, and 11 addressed only a single theme. Nineteen articles had high- to very high-quality, 21 had medium quality, and 18 had low- to very low-quality. Findings in the inputs category showed wide-ranging portal designs; patients’ privacy concerns and lack of encouragement from providers were among portal adoption barriers while information access and patient-provider communication were among facilitators. Several methods were used to train portal users with varying success. In the processes category, sociodemographic characteristics and medical conditions of patients were predictors of portal use; some patients wanted unlimited access to their EMRs, personalized health education, and nonclinical information; and patients were keen to use portals for communicating with their health care teams. In the outputs category, some but not all studies found patient portals improved patient engagement; patients perceived some portal functions as inadequate but others as useful; patients and staff thought portals may improve patient care but could cause anxiety in some patients; and portals improved patient safety, adherence to medications, and patient-provider communication but had no impact on objective health outcomes.

**Conclusions:**

While the evidence is currently immature, patient portals have demonstrated benefit by enabling the discovery of medical errors, improving adherence to medications, and providing patient-provider communication, etc. High-quality studies are needed to fully understand, improve, and evaluate their impact.

## Introduction

The increasing adoption of electronic medical records (EMRs) by hospitals presents an opportunity for patients to access their clinical data and actively participate in their care via the EMR. Hospitals and other health care organizations can facilitate patient access to their EMR information through patient portals. Patient portals can provide secure, online access to personal health information [1] such as medication lists, laboratory results, immunizations, allergies, and discharge information [2]. They can also enable patient-provider communication using secure messaging, appointments and payment management, and prescription refill requests [2,3].

The increase in patient portal implementation is, in part, due to some preliminary evidence that they may improve patient engagement [4] and health outcomes such as medication adherence [5-10]. Government incentive programs and regulations also influenced some health care organizations to implement patient portals [11,12]. For example, in the United States, implementing patient portals was a way to meet the requirements for Meaningful Use, Stage 2, of the Healthcare Information Technology for Economic and Clinical Health Act [13].

Promoting patient involvement in health care delivery may lead to improved quality and safety of care [14,15] by enabling patients to spot and report errors in EMRs, for example [6]. Some patients recognize the role of patient portals in their health care, reporting satisfaction with the ability to communicate with their health care teams and perform tasks such as requesting prescription refills conveniently [3,16]. Portal use may reduce in-person visits, visits to emergency departments, and patient-provider telephone conversations [3,8-10,12,16]. Despite the potential of portals, already used in the ambulatory setting for some time, implementation in the inpatient setting has only recently gathered momentum [17-19]. The inpatient setting presents additional challenges for implementing patient portals [18,20]. Clinical conditions leading to hospitalization are often acute and the amount of medical information generated during this time can be extensive, which may overwhelm patients [20] and challenge information technology to rapidly display this information.

The aim of this study was to review literature describing patient portals tethered to an EMR in inpatient settings, their role in patient engagement, and their impact on health care delivery in order to identify factors and best practices for successful implementation of this technology and areas that require further research. Our review aims to inform researchers, health care organizations, and policymakers.

## Methods

### Search Strategy

The PubMed, CINAHL, and Embase databases were searched for articles published between 2005 and 2017 using keywords related to patient engagement, electronic health records, patient portals, and their associated subject headings in each database: the full search terms for each database are provided in Multimedia Appendix 1.

### Study Selection and Quality Assessment

**Figure 1:**
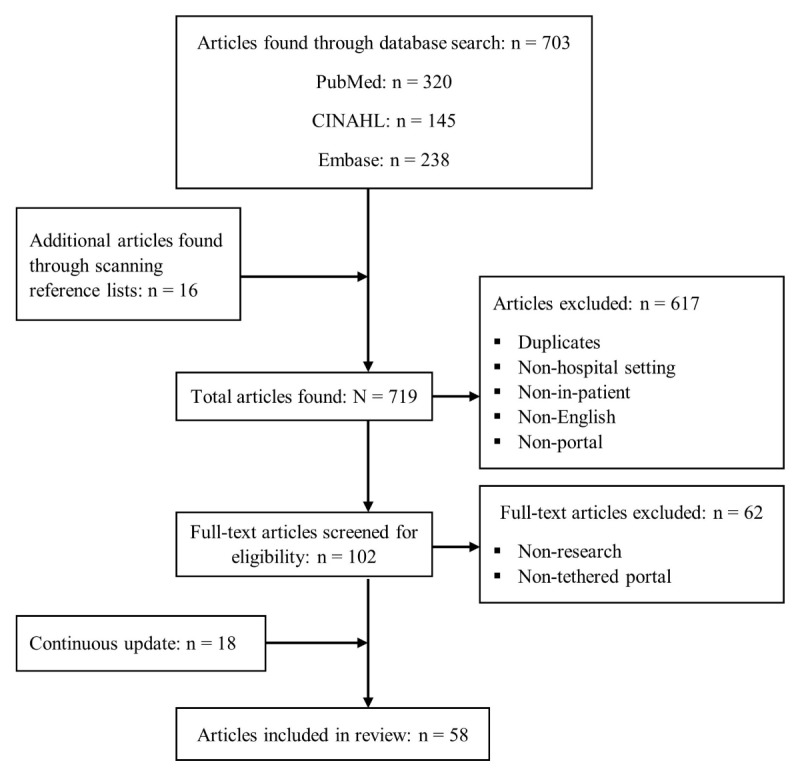
Preferred Reporting Items for Systematic Reviews and Meta-Analyses flowchart of the search and selection process.

Figure 1 shows the Preferred Reporting Items for Systematic Reviews and Meta-Analyses flow diagram for the systematic search and selection process. Inclusion criteria for articles were (1) written in English, (2) hospital inpatient setting, and (3) patient portals tethered to a hospital EMR. The initial combined database search produced 703 articles, and an additional 16 were identified by scanning their reference lists. After eliminating duplicates, the article abstracts were independently reviewed by three authors to identify articles that did not meet the inclusion criteria. This led to 617 articles being excluded. Full-text screening was conducted for the remaining 102 articles, leading to the identification of 62 articles that did not meet the inclusion criteria. At each stage, the authors met to reconcile, by consensus, any disagreements about article inclusion. An independent coder also coded the 102 articles for inclusion/exclusion using our criteria, and interrater agreement was high (Cohen kappa=.75). In cases of disagreement, we opted to include the article if it addressed a potentially important policy issue (eg, privacy issues, rural/urban divide). To ensure we included as many up-to-date papers as possible, we periodically conducted database searches for new articles after the initial search. This step was performed by just one author because by this stage, the authors had established a well-developed understanding of the inclusion/exclusion criteria. This periodic update, up to August 2018, identified another 18 articles for inclusion.

The included articles were assessed for quality. Two authors independently scored each article’s quality using the most recent version of A Measurement Tool to Assess Systematic Reviews (AMSTAR 2) [21] for review articles and the Quality Assessment Tool for Studies with Diverse Designs (QATSDD) [22] for qualitative, quantitative, and mixed empirical studies. We classified the overall AMSTAR 2– or QATSDD-derived score for each paper on a 5-point scale (very low, low, medium, high, very high), thus establishing the qualities of all articles on one scale. The ratings by the authors and the independent coder were highly correlated (*r*=.81). A third coauthor then independently reviewed and reconciled the scores. We assigned valence ratings to each article to characterize the overall findings in each article regarding patient portals as positive, negative, or mixed [23].

### Data Analysis

We analyzed the information extracted from the included articles by categorizing the themes related to the implementation of patient portals into inputs, processes, and outputs. The inputs are the material (eg, hardware and software) and nonmaterial (eg, leadership) components that facilitate or impair the establishment or use of the portal. Processes include the interactions of the users with the portal. Outputs comprise the results of the implementation or the use of the portal. Through the analysis, we identified 14 themes within these three categories, shown in Textbox 1.

**Description of the themes identified in the implementation of inpatient portals.**

- Input themes
  - Portal design: umbrella term for all design-related aspects of the portal including portal interface, content, features, and functions
  - Usability: extent to which a patient portal has the property of being able to be used by patients, caregivers, and health care teams to enhance patient engagement with effectiveness, efficiency, and satisfaction
  - Barriers: factors that hinder widespread adoption or portal use
  - Facilitators: factors that motivate or enable users to sign up for or actively use a portal
  - User training: equipping patients and health care team members with the necessary skills and knowledge to effectively use a portal
  - Organizational factors: culture of a health care organization; decisions and actions it takes when an initial consideration is made to implement a patient portal
- Process themes
  - Adoption: from a patient perspective, adoption is the registration for a portal account; from a health care provider perspective, portal adoption refers to acceptance and promotion of the portal [16]
  - Use: active engagement and continued use after signing up for a portal [4]
  - Information: all aspects associated with providing patients with clinical and nonclinical information via a portal
  - Communication: all aspects associated with portal-based patient-provider communication
- Output themes
  - Patient engagement: active involvement of patients in their own health care
  - User perceptions: thoughts, feelings, and opinions of patients, caregivers, and health care team members about their experiences with a patient portal
  - Health outcomes: impact of patient portals on clinical indicators
  - Benefits: value provided by patient portals to the health care delivery process

## Results

### Overview: Search Strategy, Study Selection, and Quality Assessment

Details of the 58 articles included in this review are given in Multimedia Appendices 2 and 3. The majority of the articles (41) are indexed in all three databases; 13 articles are indexed in two databases, and only 4 are in indexed in a single database. The PubMed and Embase databases each index 56 articles while CINAHL indexes 41 articles. Nineteen articles described qualitative studies, 18 quantitative studies, and 9 mixed method studies, while 12 were reviews. Our quality assessment placed 19 articles in the high- or very high–quality categories, 21 in the medium category, and 18 in the low- or very low–quality categories. Twenty-nine articles were assigned mixed valence, 16 articles were assigned positive valence, and 2 were assigned negative valence. Valence was not applicable or could not be drawn in 11 articles (ie, those that examined portals but that were not focused on evaluating them in any way).

There was a spread of articles addressing themes over the inputs, processes and outputs categories (see Multimedia Appendix 3). However, only one-third of the articles (19/58) addressed the full spectrum of categories.

Textbox 2 shows that there was corroboration among studies for some findings (eg, information) but contradicting findings in some themes (eg, health outcomes). We did not observe trends in the findings that were related to study quality or design (ie, there was no association between study quality or study design and reported findings).

**Summary of findings in the reviewed literature.**

- Inputs
  - Portal design
    - Portals were designed using an iterative approach [6,16,24]
    - Integrated infobuttons linked users with reliable sources of medical information [19,25-27]
    - Artificial intelligence was used to enhance portal designs [28]
    - Portals can be designed for specific diseases or medical conditions [29]
    - Patients requested electronic games within portals [18,30] and functionality to control caregivers’ access to their electronic medical records (EMRs) and receive notifications when their EMRs had been viewed by a caregiver [31]
  - Usability
    - Some participants had difficulties using patient portals mainly because of complex portal interfaces [19,24,32-34]
    - Some patients found patient portals easy to use [17,30,35-37]
  - Barriers
    - Lack of appropriate training [33]
    - Doubt of the portal’s usefulness [38]
    - Lost passwords [38,39]
    - Difficulties in using portals [16,33,34]
    - Anxiety associated with viewing personal medical information [38]
    - Data security and privacy concerns [34,40]
    - Lack of encouragement from providers [3,40]
  - Facilitators
    - Access to information [18,19,24,30,36,40-42]
    - Patient-provider communication [26,34,40,43]
    - Record-keeping [36,42,43]
    - Provider encouragement [40]
  - User training
    - Patients were trained using videos and reading material [24,33]
    - Health care teams were trained using verbal instructions and hands-on sessions [5,30,44,45]
  - Organizational factors
    - Rural hospitals were more likely to report costs and obtaining staff cooperation as barriers to health information technology (HIT; including patient portals) adoption than urban hospitals [46]
    - Small hospitals were more likely to report cost-related barriers than large hospitals [46]
    - Leaderships had crucial roles in the implementation of patient portals, working closely with developers in system design, developing policies to guide user training, and integrating portals into clinical workflows [33]
    - Implementation of patient portals varied across organizations due to different interpretations of government legislations by the health care organizations [47]
- Processes
  - Adoption
    - Portal use was higher among white patients than other racial groups, younger patients than older patients, female patients than male patients, and high-income than low-income patients [4,6,33,48]
  - Use
    - Portal use was higher among patients with greater disease severity [4]
    - Patients were less inclined to use a portal when they were seriously ill, in intense pain,
      or soon after undergoing multiple tests or procedures [24]
  - Information
    - Patients wanted timely and comprehensive access to their medical information [6,18,24,26,30]
    - Some patients preferred to have access to their entire EMR, including doctors’ notes [24,41]
    - Patients wanted personalized information tailored to their conditions and needs [6,24,35,49]
    - Patients requested clinical unit maps, meal menus [30], and short biographies of their health care team members [19,24]
  - Communication
    - Patients and caregivers expressed interest in using portals to communicate with health care staff [6,24,30,36] but not many actually used this feature [30,44]
    - Patients used portal messaging to request information, communicate needs and concerns, contribute to care coordination, offer feedback [26], compliment health care staff, and express gratitude [30,45]
    - Some patients wanted an option to send messages to specific staff members and an indication of whether a message had been read and when to expect a response [33]
    - Ethnicity, age, and gender were associated with portal-based communication [7,16,33,48]
- Outputs
  - Patient engagement
    - Some portals did not significantly improve patient engagement [17,50] but others did [6,41,43,51,52]
    - Patients in some studies reported that portals enabled better engagement in their own care [19,24,30]
  - User perceptions
    - Some patients felt that portals did not adequately fulfill their information needs [6,24,29,42]
    - Patients associated unrestricted access to their EMRs with empowerment and a sense of control [24,36,42,53]
    - Some patients and health care staff had concerns that unrestricted access to sensitive information may cause anxiety and more questions for health care staff [24,41,49]
    - Health care teams had preimplementation concerns about disruptions to workflows and potential for large volumes of patient messages, but such concerns did not materialize [45]
    - Patients and staff thought that the messaging feature of patient portals was important for patient care [17,24,26,30]
    - Patients who used a disease-specific portal were more satisfied than those who used a generic portal [29]
    - Patients and health care staff agreed that patient portals helped to improve patient care [5,30,44]
  - Health outcomes
    - Significant association between portal use and health outcomes was not observed in some studies [2,4,20] but was observed in others [54]
    - Patient portals facilitated discovery of EMR errors by patients [24,26,30,54-56]
  - Benefits
    - Improved adherence to medication [3,9,39,43,52,57]
    - Improved patient satisfaction [3,29,30,39,45]
    - Enhanced patient-provider communication [6,30,40,43,52]
    - Improved patient safety [6,26,52,55]
    - Reduced patient uncertainty and anxiety [3,24,36]
    - Increased patient engagement [6,19,24,30,41,43,45,51,52]

### Patient Portal Inputs

Forty articles addressed themes in the inputs category. We identified 22 articles that addressed portal design. In an iterative design approach, feedback from patients, including requests for electronic games and other functions, was used to refine designs. Enhancements included links to medical education, artificial intelligence techniques, and disease-specific design.

As shown in Textbox 2, poor portal designs caused usability difficulties for some patients. Those difficulties were among the barriers to portal use. Textbox 2 also shows that more barriers to portal use than facilitators were identified in the reviewed literature. Various methods were used to train patients and staff to use the portals. However, training methods were not optimal [5,33,44]. For example, in one study, patient training was delivered via an 11-minute video that was not well received, with less than a third (26.3%) watching the entire video [33]. In another study among health care staff, doctors had the lowest confidence in a patient portal, and they doubted their training was sufficient to allow them to effectively use it [5].

Organizational factors (leadership, staff support, and key decisions, etc) was the least addressed theme. However, the findings summarized in Textbox 2 indicate that organizational factors are likely the most crucial in determining whether or how patient portals are implemented.

### Patient Portal Processes

Thirty-six articles addressed themes categorized as processes. Five articles addressed portal adoption while use was addressed in 18 articles (see Multimedia Appendix 3). Sociodemographic characteristics of patients such as race, age, gender, level of education, and social status were predictors of both portal adoption and use [4,6,33,48]. In addition to sociodemographic factors, there was higher portal use among patients with greater disease severity (eg, advanced cancer) [4], but at any given time, patient condition influenced portal use as described in Textbox 2.

Seventeen articles addressed information and 20 addressed communication. The articles suggested that patients wanted unlimited access to their EMRs, medical education in layman’s terms [24,41], and nonclinical information [19,24,30].

Patient-provider communication, usually in the form of secure messages, is a key feature of inpatient portals [26]. Despite expressions of interest in this feature, actual use was low [30,44]; in a pediatric study with 296 parents participating, only about 6% sent messages to health care teams via the inpatient portal [30]. Most portals enabled patients to send messages to a single mailbox that was accessed by staff members on duty, but some patients wanted to communicate with specific staff members [33]. Similar to adoption and use, sociodemographic factors were predictors of which patients used the messaging feature (see Textbox 2).

### Patient Portal Outputs

The outputs category was addressed by 46 articles, and 24 articles addressed patient engagement. Results of patient engagement were mixed: portals in some studies did not cause statistically significant improvement, but patients in other studies reported that portals enabled better engagement in their care.

User perceptions was the most commonly addressed theme across all categories (35 articles), and Textbox 2 shows that users perceived some functions of portals (eg, access to information) as inadequate but perceived other functions (eg, communication) as very useful. Despite dissatisfaction and concerns with some aspects of the portals, users’ perceptions of the patient portal concept as a whole were mostly positive. For example, in one study, 90% of participants reported overall satisfaction with the portal, 89% thought that the portal reduced errors, and 94% agreed that the portal improved care delivery [30]. In another study, 84% of participants described a patient portal as useful and 90% reported that they would recommend it to their peers [32]. Health care staff appreciated the reasons for implementing patient portals and their own roles and responsibilities in the process, but they stated that they would like to receive sufficient training [5].

Twenty articles addressed the association between patient portals and health outcomes, such as medical errors, readmissions, and mortality. Results were mixed as some studies did not show positive associations between portal use and health outcomes [2,4,20]. For example, a retrospective study of cancer patients found no association between portal adoption or use and adverse events [4] and another retrospective study found no association between portal use and 30-day readmission or inpatient mortality [20]. However, in a third study, active portal use significantly improved glycated hemoglobin levels [54]. Importantly, patient portals in several studies facilitated discovery of EMR errors by patients, particularly medication errors [24,26,30,54-56].

Finally, 7 articles addressed the benefits of patient portals, and Textbox 2 shows that portals offers a wide range of benefits for patient care.

## Discussion

### Principal Findings

**Figure 2:**
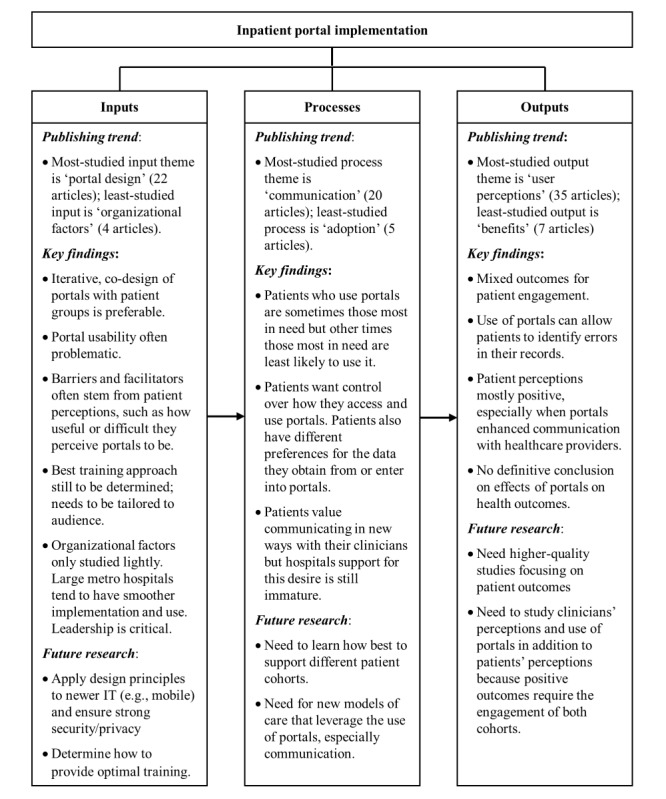
Conceptual framework summarizing the findings and key areas for future research.

This systematic review examined 58 articles studying inpatient portals. Although there was overlap in the themes reflected in these studies, there was also significant variation in the setting, patient population, software, outcomes assessed, and study methodology, making it hard to come to a definitive conclusion on whether inpatient portals are beneficial. This is further shown by the higher number of included studies that were judged to relay mixed valence than those with positive valence. However, more studies relayed positive than negative valence, showing that patient portals may be beneficial for health care. We discuss below the patient portal input, process and output factors that contribute to this assessment, and the areas of research that need focus in order to improve patient care. Figure 2 provides a visual summary.

### Patient Portal Inputs

When assessing common themes within the inputs category, portal design, usability, and barriers were more widely covered than user training and organizational factors. Many articles showed that good portal design is crucial for usability and adoption by patients. While most studies involved users in the design process, more needs to be done to overcome design-related barriers, particularly for people with low health literacy [6,19,24]. Increasing the use of enabling technology such as voice-commanded digital assistants, artificial intelligence, and natural language processing could make the systems cater to a wider range of audiences. Designing platforms so that they can be displayed on users’ personal mobile devices could be another enabler of portal adoption.

Patients’ concerns regarding privacy and security of their medical information [34,40] are particularly relevant in cases where caregivers need access to patients’ EMRs. Discussion of privacy issues in cases of caregiver access to patient medical information in the literature is limited. In the absence of formal policy for caregiver access, patients may opt to disclose personal log-in details to their caregivers, which is not recommended [26].

The results of user training showed that reliance on a one-size-fits-all approach may not be effective for educating users because of varying preferences. Training could be enhanced by providing information that directly addresses common patient concerns (eg, information security) and health care staff concerns (eg, workflow changes).

Information on the best timing to deliver training material to patients is lacking in the literature. While staff training can be scheduled ahead of deploying a patient portal, patients may only encounter a portal upon hospitalization. Furthermore, the severity of their condition may limit their ability to focus on or understand the training material. Innovation may be needed to inform patients about portal services prior to hospitalization. For example, patients could sign up for outpatient portals which would be similar in design to the inpatient portals to ensure seamless transition between the two portals. Hospitals could also collaborate with medical insurance providers to make training material available to potential patients already signing up for hospital insurance.

### Patient Portal Processes

Associations between patient sociodemographic characteristics and portal use [4,6,33,48] indicate that patients who are most vulnerable (eg, those with low health literacy or seriously ill) would be least likely to benefit from patient portals. Recruitment and participation bias when engaging participants in user testing mean that actual use by patients may not reflect the use in the initial testing phases. Therefore, health care organizations may need to conduct multiple studies to iteratively address factors that influence portal use within the communities they serve.

The varying preference among patients for level of access to EMRs [6,18,24,26,30] and staff concerns with unlimited patient access to sensitive information (information that could cause anxiety for patients) [24,41,49] present challenges for health care organizations. Health care organizations could fulfill these diverse information needs by flagging sensitive information and warning patients that despite having access to the information, they can opt to receive it in person from a health care team member. Some hospitals already have guidelines for releasing sensitive information to outpatient portals [26], and those guidelines could be adapted to the inpatient setting.

Health care organizations may view patient requests of nonclinical information and functions such as electronic games [18,19,24,30] as fringe requests that could raise developmental costs. However, granting these requests may improve portal adoption and use and overall patient satisfaction. Also, patients have requested background information on their health care staff, but staff perceptions about the disclosure of such information to their patients is not known [19,24]. Further research is needed to uncover staff perceptions in this regard and explore ways of implementing this service.

Portal-based patient-provider communication is potentially beneficial but may also be disruptive [26]. Although staff concerns about potential interruptions caused by constant patient messaging did not materialize [45], in practice, staff members may be overwhelmed by messages at any time. Structured messaging may be a solution that ensures patients communicate only important and relevant information [26]. If health care organizations decide to enforce structured messaging, they should prioritize patient safety and therefore avoid restrictions that could prevent patients reporting genuine concerns.

### Patient Portal Outputs

The literature explored some, but not all, potential outputs of patient portal implementations. Most of the studies assessed implementation of patient portals using interim outcomes such as user perceptions, and few studies addressed important objective outcomes such as length of stay, morbidity, or mortality [2,4,20,54].

Some studies showed no association between portal use and health outcomes such as readmission, adverse events, or mortality [2,4,20]. However, a number of those studies drew their conclusions from retrospective analysis of portal adoption and use data only [4,20]—adoption and frequency of use alone do not provide sufficient information about effective portal use, which may affect outcomes.

Increasing patient engagement is a goal of patient portals, but the engagement of health care staff is also important since they are likely to be approached by the patients with portal-related queries [5,44]. Nurses and doctors should have sufficient knowledge to answer basic questions or appropriately escalate complex questions (eg, to information technology support). Also, nurses may be required to respond to patient-generated messages within the portal. Similarly, doctors’ perceptions of portals are also important, as they may use a portal to communicate with patients [5] and therefore need to be confident with its functions. Theories in health care information technology suggest that user perceptions can predict the acceptance and use of new technologies [58-60]. Therefore, it is essential for hospitals to ensure positive staff attitudes toward patient portals through effective staff training and technical support and incorporating staff needs in portal design and workflows.

Several studies reported that patient portals facilitate patient discovery of errors in EMRs [24,26,30,54-56]. Discussion in the literature of how patients could notify health care providers of such errors is lacking. Further research is needed to establish how patient-discovered errors are reported and to identify optimal reporting methods.

Research in the evaluation of patient portals is also currently limited. Standardized evaluation frameworks and measures are needed to enable better comparisons of patient portal implementation and outcomes in the future.

### Limitations

While an extensive search was undertaken, the majority of the included studies were conducted at single locations, used outcome measures that were not comparable to those used in other studies, and had small sample sizes. That means results of those studies may not be generalizable to other population groups. Also, a number of the studies were conducted in controlled settings, such as closed-door observations and interviews, which would not be representative of hospital settings. The absence of standardized evaluation tools means the results could not be compared or synthesized, and we were thus limited to providing a descriptive summary of findings only. Most studies that addressed user perceptions or patient-reported results depended on the opinions of those who completed end-of-study questionnaires or interviews; such results could be biased as they may lack feedback from participants who felt uneasy about giving negative feedback. Finally, our conceptual framework enabled us to group the findings gleaned from the included articles, although there was some overlap in the categories caused by interdependence in some of the themes.

### Conclusion

The review results suggest that the available evidence for inpatient portals is currently immature. Standardized outcomes assessment and more high-quality studies with objective outcomes (length of stay, mortality, and morbidity) are required to fully understand the impact of such portals.

## Appendix

Multimedia Appendix 1 Search terms for the PubMed, CINAHL, and Embase databases.

Multimedia Appendix 2 Detailed summary of each article selected for inclusion in the review.

Multimedia Appendix 3 Themes addressed by each article.

## Abbreviations

AMSTAR: A Measurement Tool to Assess Systematic Reviews
EMR: electronic medical record
HIT: health information technology
QATSDD: Quality Assessment Tool for Studies With Diverse Designs
