# Supplementary material for: Patient Portals Facilitating Engagement With Inpatient Electronic Medical Records: A Systematic Review
Source: J Med Internet Res. 2019 Apr 11;21(4):e12779. doi: 10.2196/12779 (PMC6482406; doi:10.2196/12779)
Supplement: Multimedia Appendix 1 [file jmir_v21i4e12779_app1.pdf]

Multimedia Appendix 1: Search terms for the PubMed, CINAHL and Embase databases.

PubMed

Keywords: patient engagement, consumer engagement; electronic health records, electronic medical records, patient portal

MeSH terms: patient participation, electronic health records, patient portals

PubMed Search:

(("patient participation"[MeSH] OR "consumer engagement" OR "patient engagement")) AND ("electronic health records"[MeSH] OR "electronic health records"[tiab] OR "electronic medical records"[tiab] OR "patient portals"[MeSH] OR "patient portals"[tiab])

CINAHL

Keywords: patient engagement, consumer engagement, patient participation, electronic health record, electronic medical record, patient portal

CINAHL Headings: consumer participation, electronic health records

CINAHL search:

((MH"consumer participation") OR "patient engagement" OR "consumer engagement") AND ((MH"electronic health record") OR "electronic health record" OR "electronic medical record*" OR "patient portal")*

Embase

Keywords: patient engagement, consumer engagement, patient participation, electronic health record, electronic medical record, patient portal

Emtree terms: Electronic health record, electronic medical record, Patient participation,

Embase search:

(('patient engagement'/exp OR 'patient participation'/exp OR 'patient engagement' OR 'patient participation' OR 'consumer engagement') AND ('electronic health record'/exp OR 'electronic health record':ti,ab OR 'electronic medical record*':ti,ab OR 'patient portal':ti,ab))*

Abbreviations

MeSH: Medical Subject Heading

tiab: Title/Abstract (PubMed)

ti,ab: Title/Abstract (Embase)

MH: Medical Heading
