# Supplementary material for: Patient Portals Facilitating Engagement With Inpatient Electronic Medical Records: A Systematic Review
Source: J Med Internet Res. 2019 Apr 11;21(4):e12779. doi: 10.2196/12779 (PMC6482406; doi:10.2196/12779)
Supplement: Multimedia Appendix 2 [file jmir_v21i4e12779_app2.pdf]

Multimedia Appendix 2: Detailed summary of each article selected for inclusion in the review.

Author and publication year	Study type	Quality	Sample	Findings	Valence[a]		
					P	M	N
Adler-Milstein et al. 2017 [24]	Quan	Very high	Survey responses from CEOs or delegates from 2803 acute care hospitals in USA between 2015-2016	Patient portals are being used more by large urban hospitals, and less by critical access hospitals (those that are smaller and the only provider in their region).		x	
Aljabri et al. 2018 [4]	Mixed	Very high	4,594 cancer patients (2,352 adopters and 632 active users)	Portal adoption and active use was influenced by predisposing and enabling factors; active use was also influence by need (e.g. disease severity). There was higher adoption among those with more frequent hospitalizations. There was no significant difference in adverse events between portal adopters and non-adopters.		x	
Ammenwerth et al. 2012 [9]	Review	High	4 studies	There was no significant difference between intervention and control groups in 2 randomized controlled trials investigating the effect of patient portals on endpoints measuring health or proxies for health outcomes. In another study, the patient portal group had the following advantages over the control group: reduction in office visits, slower increase in telephone contact, increase in messages sent, better medication management and better medication adherence.		x	
Arnold et al. 2013 [25]	Qual	Very low	Participants at an international radiology conference (sample size not specified)	Participants supported the idea of the patient portal for radiology patients but also had concerns about patients misunderstanding results and how best to disclose information to them.		x	
Baudendistel et al. 2015 [26]	Qual	Medium	10 focus groups (n = 47), including colorectal cancer patients, patient support group members, physicians, and other health professionals.	Potential users of a personally controlled electronic health record viewed such a system positively. Patients emphasized the need for them to have responsibility as a gatekeeper and access manager.	x		
Borbolla et al. 2014 [27]	Quan	Very low	80,000 patients. The number of those patients using the Infobuttons is not reported.	When infobuttons were made available to patients, the patients clicked on infobuttons to access general health information from MedLine Plus, local information in their health record, and food recipes.	NA	NA	NA
Caine et al. 2014 [28]	Mixed	Medium	30 patients	Patients provided advice on how to design a portal to control who has access to their data. Most patients did not know what was in the EMR or who could access it, but they wished they could access it and control others' access.	NA	NA	NA
Dumitrascu et al. 2018 [20]	Quan	Very high	17,050 patients. 7,538 had portal accounts, 9,512 no portal accounts.	No significant association of portal use with inpatient mortality, 30-day readmission or 30-day mortality. Only 20.8% of those who had a portal account accessed it during hospitalization.		x	

Author and publication year	Study type	Quality	Sample	Findings	Valence[a]		
					P	M	N
Essen et al. 2017 [29]	Qual	Medium	14 policy-makers and providers	Even though the national government and health providers have the same view of the potential of patient portals, these portals are rarely implemented and used; the government and providers have very little engagement or consistency regarding such initiatives.		x	
Giardina et al. 2014 [30]	Review	Medium	27 studies	The impact of providing patients with access to their health records is equivocal for effectiveness and patient-centredness. Mixed outcomes for efficiency. Some benefits for patients' perceptions of control.		x	
Grant 2006 [31]	Quan	Very low	NA (Proposal only)	Designed and implemented a diabetes patient portal that links to a hospital EHR, which could benefit patients and primary care physicians, but not yet tested.	NA	NA	NA
Greenberg et al. 2016 [32]	Quan	Medium	33,749 patients	Compared to urban patients, rural patients are just as likely to have a health provider with an EHR, but are less likely to have regular internet access, to manage personal health information online or to email their health care providers.	NA	NA	NA
Griffin et al. 2016 [2]	Quan	Very low	2,975 patients	83% of eligible users chose not to use the portal made available to them from the health system. The most commonly used feature was the messaging feature. More active portal users were more likely to be readmitted within 30 days.		x	
Grossman et al. 2018 [33]	Qual	High	NA	Staff fear constant interruptions and overwhelming amounts of patient contact. Patients want informative and fast responses, but also fear overburdening providers. Some organizations impose structure on patient communications by asking specific questions. Patients and caregivers valued patient-provider communication and care plan features. Visual displays of laboratory results facilitate better understanding compared to tables. Access to medical notes helped to engage patients in their care. Patients preferred educational material tailored for disease, age and health literacy. Portal use can improve patient safety. Caregiver access to patient information is just as important as patient access. Information about amenities can enhance patient hospital experience.		x	
Hazara & Bhandari 2015 [34]	Mixed	Medium	69 patients	Even though patients could access the internet and use computers, they did not use the patient portal because they lost their passwords (45%), did not perceive the system to be useful (37%), were too busy (13%), or were anxious viewing the results from home (10%).	NA	NA	NA

Author and publication year	Study type	Quality	Sample	Findings	Valence[a]		
					P	M	N
Hefner et al. 2017 [5]	Qual	Medium	686 healthcare providers, 193 doctors; 439 nurses and 186 clinical support staff.	Staff had a moderately positive attitude towards the patient portal, clinical support staff were the most positively oriented towards the patient portal, followed by nurses and doctors last. Doctors were the least confident about the use of the patient portal and least satisfied with the level of training.		x	
Hefner et al. 2018 [35]	Qual	Medium	220 care staff, 4 IT staff.	Staff should be trained how to teach patients to use portals, to promote portal use among patients and to optimize patient-provider messaging. There was no standardized workflow for responding to patient messages sent to a single mailbox (i.e. which staff member should respond and when etc.) and some team members had little experience with the messaging feature. Use of portal messaging was infrequent.			x
Heyworth et al. 2014 [36]	Quan	Low	60 patients	A secure messaging tool was tested to allow patients and providers to conduct medication reconciliation through a patient portal. This helped them identify medication discrepancies in the records of 40 of 60 patients, including 23 potential adverse drug events. 90% of the patients said they would use the system again.	x		
Irizarry et al. 2015 [16]	Review	Very low	120 studies	Patients' interest and ability to use patient portals is affected by individual factors (age, ethnicity, education, health literacy, health status, caregiver status) plus provider endorsement and system usability.	NA	NA	NA
Johansen & Henriksen 2014 [37]	Review	Very low	56 studies	The literature shows some positive effects of personal health records for patient self-management but the evidence is sparse. Most personal health records do not support self-management sufficiently.		x	
Kaziunas et al. 2016 [38]	Qual	Medium	17 patients	New systems could help patients and care-givers: 1) navigate the health system (e.g., to know the clinical trials they enrolled in and who their care team is), 2) manage daily challenges of caregiving (e.g., to know lab test results), and 3) transition from inpatient care to outpatient management (e.g., to understand medication plans and how to care for the central line).	NA	NA	NA
Kelly et al. 2017a [39]	Mixed	Very high	296 parents in paediatric hospital.	Parents given a tablet-based in-hospital patient portal generally used the portal and perceived it positively (ranging from 60-98%) for satisfaction, improvement in care, and improvement in communication.	x		

Author and publication year	Study type	Quality	Sample	Findings	Valence[a]		
					P	M	N
Kelly et al. 2017b [40]	Qual	Very high	94 (out of 100) staff members completed pre-implementation survey. 70 (out of 80) completed the post implementation survey. Nurses, attending doctors and resident doctors.	Pre-implementation, staff members were optimistic about the portal capability at improving communication but less optimistic about communication with doctors. They were also less optimistic about how the portal would fit into their work and were concerned about increased workload. Respondents anticipated challenges using the portal such as high volume of questions. There was lower agreement post-implementation than at pre-implementation that portal use improved communication. Fewer challenges reported at 6 months after implementation. Respondents reported increased parent satisfaction, access to information, recognition of staff and better engagement as the best thing about the portal.	x		
Kelly et al. 2018 [6]	Review	High	17 studies	Patients require more information and those needs can influence portal design. Patients value transparency and have interest in communication via portals. Patients prefer simple interfaces. Most patients and caregivers found portals easy to use, useful and expressed satisfaction with their usability. There was high usage of the portals' messaging function. Mixed results for portal effect on patient engagement and knowledge. Portals can help improve patient-provider communication and facilitate the discovery of medical errors by patients. Providers have concerns with transparency in information provision.		x	
Klein et al. 2017 [41]	Quan	Medium	620 patients and 133 healthcare providers	Patients and providers evaluated a new portal feature from Veteran's Affairs. Most patients (78%) thought that the information would help them to become more involved in their healthcare. Provider evaluations ranged from 50% (improved testing) to 97% (improved information accuracy).	x		
Kruse et al. 2015 [3]	Review	Medium	27 studies	Most studies of the impact of patient portals on chronic-condition outcomes only examined some features, not the entire portal. There was limited evidence of benefits on medical outcomes, but showed some benefits for patient satisfaction, retention, knowledge, and medication adherence.		x	
Lee et al. 2017 [42]	Quan	Low	50 (33 women, 38 guardians, 40 Android users) in portal group and 52 (37 women, 34 guardians, 44 Android users) in app group	A quasi-experiment that tested the difference between providing patients with a *generic* portal (for any disease) or a *specific* portal for their condition found that both types of systems helped increase patients' knowledge but that satisfaction was greater in the specific case.	x		

Author and publication year	Study type	Quality	Sample	Findings	Valence[a]		
					P	M	N
Mikles & Mielenz 2014 [43]	Quan	Low	5,622 patients.	Multiple demographic, socioeconomic, and health-related patient characteristics were associated with the use of the portal. Greater use was associated with being female, receiving more messages from the health provider, and having a high socioeconomic status.	NA	NA	NA
Nazi et al. 2013 [44]	Quan	Low	688 US Veterans.	84% of patients agreed the information and services made available to them from the VA EHR was helpful. 72% indicated that the pilot website made it easy for them to locate relevant information. 66% agreed that the pilot helped improve their care, with 90% indicating that they would recommend it to another veteran.	x		
O'Leary et al. 2016 [17]	Quan	Very high	202 patients; 100 with with the portal, and 102 without tablet or portal.	Patients receiving a tablet-based portal were more able than patients not receiving the portal to name the physician caring for them but had no greater activation in the care process and no greater knowledge of the nurse caring for them, the care plan, or their medication status.		x	
Osborn et al. 2010 [45]	Review	Low	26 studies	Web portals can have a range of positive patient outcomes for diabetic patients, including perceptions (e.g., satisfaction) and medical outcomes (e.g., disease management, status), but many of the portals have unique aspects, so the aggregate results must be interpreted carefully. More work needed on which functionalities help, how best to use them, and what processes need to be supported.	x		
Otte-trojel et al. 2016 [7]	Review	Low	109 studies	Identified five main problem categories with patient portal development: 1. patient engagement, 2. provider engagement, 3. data governance, 4. security and interoperability, and 5. a sustainable business model. The review identified causes of and solutions to these problems, but very few studies actually implemented and evaluated the solutions, so their usefulness is unknown.	NA	NA	NA
Phelps et al. 2014 [46]	Quan	Very high	11,352 patients.	A large proportion of patients regularly used the patient portal and liked detailed information about their care.	x		
Pillemer et al. 2016 [47]	Mixed	Very high	14,441 patients.	Patients valued access to test results through the portal and it increased their engagement, but access could increase patient anxiety and increase patient visits.		x	
Powell 2017 [48]	Review	Very low	37 studies.	Patient portals have different effects depending on the users involved and the presence of facilitators (provider encouragement, data access/control, and enhanced communication) and barriers (lack of awareness/training, and privacy/security concerns).		x	

Author and publication year	Study type	Quality	Sample	Findings	Valence[a]		
					P	M	N
Prey et al. 2014 [18]	Review	Medium	17 studies.	The review showed that there needed to be more research on inpatient engagement technologies, particularly in terms of impact on health outcomes and cost-effectiveness.	NA	NA	NA
Prey et al. 2016 [49]	Qual	Medium	16 participants.	Patients around the world are being provided with access to their data. But there is considerable variability in the level of maturity of the patient engagement/involvement concept, the degree of Government involvement, technical infrastructure, and plans for future development globally.		x	
Ralston et al. 2013 [50]	Quan	Very low	3,888 patients.	Shared medical record (SMR) use was higher among patients who had greater recent need for healthcare, and lower among patients from several racial and ethnic minority groups, among women, and among patients with a history of injection drug use and lower socioeconomic status.		x	
Rappaport et al. 2016 [51]	Qual	Very low	4 senior health IT officials.	The patient portal provided patients and families with increased access but gaps in functionality remained, e.g., inability to identify patient and clinician; inability to select providers as electronic health information (EHI) recipients and update these relationships over time; complication of varying state regulations for sharing records; and reliance on some manual functions.		x	
Rathert et al. 2017 [52]	Review	Medium	41 studies.	The literature suggests that EHRs can improve information capture and sharing but can interfere with clinician-patient relationships, and that patient portals can improve communication, patient empowerment, engagement, and self-management.		x	
Rexhepi et al. 2018 [53]	Qual	Medium	30 cancer patients.	Patients use online access to prepare for doctors' visits and learn more about their disease. Patients do not appear to feel anxious reading results online, were respectful of clinician's time in following up questions, were not worried about complex information, and trusted security and privacy controls in place.	x		
Risling et al. 2017 [54]	Review	High	28 studies.	Of 19 empirical studies, only four examined patient empowerment and they all viewed the concept differently. As a result, while tethered patient portals are implemented to improve patient empowerment, the literature offers insufficient evidence of this link.		x	
Runaas et al. 2016 [55]	Mixed	Very low	10 caregivers for paediatric patients.	An iPad-based tool with personalized and real-time health information from the EHR adapted to specific clinical conditions has the potential to increase caregiver activation'. The iPad was reported as easy to use.	x		

Author and publication year	Study type	Quality	Sample	Findings	Valence[a]		
					P	M	N
Saberi et al. 2012 [56]	Quan	Medium	HIV-positive patients. Sample size not given.	Patients' use of shared medical records is associated with greater adherence to medication regime, with small declines in non-users. This is notable because prior research shows that even small declines are link to higher mortality risk.	x		
Snyder et al. 2013 [57]	Mixed	Medium	76 cancer patients; 11 clinicians.	The pilot test of the website showed that patients reported it to be easy to use (92%), useful (70%), to improve recall of symptoms/side effects (72%), to help them feel more in control (60%), to improve discussions with provider (49%), and improve care (39%). Clinicians and patients both wanted more information from the system and minor adjustments to it.		x	
Sorensen et al. 2009 [58]	Mixed	Medium	69 nursing informaticians.	16 nursing informaticians reported that their institution had a secure patient portal; 32 others had plans to develop one and 11 reported that no set time limit had been set but that long range plans were in place.		x	
Toscos et al. 2016 [59]	Quan	Very high	200 Coronary Artery Disease (CAD) patients; 184 in 6-month survey; and 173 completed 12 month survey.	Use of the patient health record did not lead to a significant improvement in patient engagement. HbA1c levels improved significantly in the *Active* and *Super* user groups at 6 months. Use of the patient health record diary increased significantly following education at the 6-month study visit and an elective group refresher course.		x	
van der Vaart et al. 2014 [60]	Quan	Very high	360 patients.	54% of respondents with internet access viewed their EMRs. They were positive about ease of use and usefulness and reported few problems. Age, amount of internet use and self-perceived internet skills significantly predicted portal use. No significant differences were found over time for measures of patient empowerment.		x	
van der vaart et al. 2013 [61]	Mixed	Very high	Phase 1: 13 rheumatologists and 9 nurse/nurse practitioners from 9 different hospitals interviewed. Phase 2: 9 rheumatologists and 8 nurses/nurse practitioners responded to questionnaire	Clinicians felt an EMR portal could improve patient participation, enhance patient knowledge and treatment adherence, build trust, improve safety, and enhance communication, but that it could also stress patients (due to misinterpretation) and add work for administrators and physicians. Suggestions included placing filters on clinical notes, not writing extra notes, adding an inaccessible section for personal notes, and controlling how and when patients receive data. Most respondents didn't want patients to access their clinical notes.		x	

Author and publication year	Study type	Quality	Sample	Findings	Valence[a]		
					P	M	N
Vawdrey et al. 2011 [19]	Qual	Very low	5 cardiology patients.	Patients reported varying levels of comfort with the tablet-based portal, but they were still enthusiastic about its potential to provide them with their medical information and details of their care teams.	x		
Walker et al. 2018a [62]	Qual	Very high	19 current and former patients.	Users had issues with the physical lay-out of the application which caused challenges in navigating the app. Some users could not understand some of the information displayed in the portal (e.g. lab results). Users expressed a need for personalized information like medication and condition-based education. Users expressed concern that the portal might place more burden on the care team by raising a lot of questions. Users were concerned that the design of the application would cause anxiety for patients		x	
Walker et al. 2018b [63]	Qual	Very high	2 focus groups of 12 and 8 stakeholders. Online forum of 14 stakeholders.	Potential evaluation measures were categorized into work system, processes and outcomes. Potential measures for work systems included, among others, usability testing, patient activation measure, self-efficacy, training, communication and leadership. Potential measures for processes included, among others, workflow analysis, frequency and intensity of use and patient learning. Potential measures for outcomes included, among others, satisfaction, use of outpatient portal and clinical indicators. Patients ranked satisfaction as the most important outcome measure while care team and hospital management ranked efficiency and quality as most important outcome measures, respectively.	NA	NA	NA
Wibe & Slaughter 2009 [64]	Qual	Very low	8 interviews.	Emotions can affect how patients read their record, e.g., finding errors in their record can lead to patient distrust. Emotional reaction can also be caused by discrepancies between oral communication and documentation. Need to consider the types of comments (positive/negative) in the record and the consistency of oral/written comments.			x
Winkelman et al. 2005 [65]	Qual	Very high	12 patients.	Patients viewed access to electronic patient records positively. Four themes emerged in the study: 1. illness ownership, 2. patient-driven communication, 3. personalized support, 4. mutual trust between clinicians and patients.	x		

Author and publication year	Study type	Quality	Sample	Findings	Valence[a]		
					P	M	N
Woollen et al. 2016 [66]	Qual	Medium	14 patients and their caregivers.	All patients responded favourably to having access to their clinical information. 85.7% used the application. Patients reported high satisfaction with being able to view their hospital medications and access to educational materials. Patients reported a desire for additional patient-focused information too in a user-friendly format.	x		
Wright et al. 2014 [67]	Quan	Medium	3,649 patients.	Overall, patients found the ability to view their problem lists via an online personal health record to be very useful and took action in response to the information. However, some had negative emotions.		x	
Yen et al. 2018 [68]	Qual	Very high	19 current and former patients.	Observers registered 224 operational errors which varied by age (participants below 40 had the lowest error count; over 60s had the highest count), 68 system errors and 9 tablet-related errors. Most participants were not interested in watching the 11-minute tutorial video because it was too long. Users preferred shorter, less detailed tutorial videos, explorative learning, in-person instructions and guided handouts. There were mixed opinions about the messaging function, users wanted to select who would receive their messages and to view the status of their messages once they have been sent.		x	
Zarcadoolas et al. 2013 [69]	Qual	Medium	4 focus groups with 28 low-education level, vulnerable English-speaking healthcare consumers	Mostly positive perceptions by focus group members- Participants from vulnerable populations see value in using patient portals and are motivated and very interested in using them.	x		
				Totals	16	29	2

Notes: Qual = Qualitative; Quan = Quantitative; NA = Not Applicable
[a]Valence: P = Positive; M = Mixed; N = Negative

Abbreviations:

CEO = chief executive officer

EHI = electronic health information

EHR = electronic health record

SMR = shared electronic medical record
