# Supplementary material for: Patient Portals Facilitating Engagement With Inpatient Electronic Medical Records: A Systematic Review
Source: J Med Internet Res. 2019 Apr 11;21(4):e12779. doi: 10.2196/12779 (PMC6482406; doi:10.2196/12779)
Supplement: Multimedia Appendix 3 [file jmir_v21i4e12779_app3.pdf]

Multimedia Appendix 3. Themes addressed by each article.

	**Category**													
	**Inputs**						**Processes**				**Outputs**			
Authors, year	Portal design	Usability	Barriers	Facilitators	User training	Organizational factors	Adoption	Usage	Information	Communication	Patient engagement	User perceptions	Health outcomes	Benefits
Adler-Milstein et al 2017 [24]						x								
Aljabri et al 2018 [4]				x			x	x					x	
Ammenwerth et al 2012 [9]										x	x	x	x	
Arnold et al 2013 [25]	x											x		
Baudendistel et al 2015 [26]												x		
Borbolla et al 2014 [27]	x													
Caine et al 2015 [28]	x											x		
Dumitrascu et al 2018 [20]													x	x
Essen et al 2017 [29]						x								
Giardina et al 2014 [30]									x		x	x	x	
Grant 2006 [31]	x													
Greenberg et al 2016 [32]						x								
Griffin et al 2016 [2]								x		x	x		x	
Grossman et al 2018 [33]	x							x	x	x	x	x	x	x
Hazara & Bhandari 2015 [34]			x				x	x				x		
Hefner et al 2017 [5]					x							x		
Hefner et al 2018 [35]					x					x		x		
Heyworth et al 2014 [36]	x		x								x		x	
Irizarry et al 2015 [16]	x	x	x	x			x	x		x				
Johansen & Henriksen 2014 [37]	x												x	
Kaziunas et al 2016 [38]	x								x		x			
Kelly et al 2017a [39]								x	x	x		x	x	
Kelly et al 2017b [40]					x					x	x	x	x	
Kelly et al 2018 [6]	x	x			x			x	x	x	x	x	x	
Klein et al 2017 [41]					x				x	x		x		
Kruse et al 2015 [3]			x	x				x				x	x	
Lee et al 2017 [42]	x								x			x		
Mikles & Mielenz 2014 [43]								x		x				
Nazi et al 2013 [44]		x						x	x			x		
O'Leary et al 2016 [17]	x										x	x		
Osborn et al 2010 [45]		x	x							x	x	x	x	
Otte-trojel et al 2016 [7]	x										x			
Phelps et al 2014 [46]							x	x			x			
Pillemer et al 2016 [47]								x	x		x	x	x	
Powell 2017 [48]			x	x				x				x		
Prey et al 2014 [18]	x								x	x				
Prey et al 2016 [49]									x					
Ralston et al 2013 [50]								x						
Rappaport et al 2016 [51]	x													
Rathert et al 2017 [52]										x	x			
Rexhepi et al 2018 [53]				x					x	x	x	x		
Risling et al 2017 [54]											x	x	x	

Runaas et al 2016 [55]		x									x	x		x
Saberi et al 2015 [56]													x	
Snyder et al 2013 [57]		x						x	x	x	x	x	x	
Sorensen et al 2009 [58]	x											x		
Toscos et al 2016 [59]								x			x		x	
van der Vaart et al 2014 [60]		x						x		x	x	x		
van der Vaart et al 2013 [61]	x								x	x	x	x	x	x
Vawdrey et al 2011 [19]	x	x							x		x	x		
Walker et al 2018a [62]	x								x			x		
Walker et al 2018b [63]	x													
Wibe & Slaughter 2009 [64]										x	x	x		
Winkelman et al 2005 [65]										x		x		
Woollen et al 2016 [66]	x	x		x	x		x	x	x		x	x		x
Wright et al 2014 [67]												x	x	x
Yen et al 2018 [68]		x	x	x	x	x				x		x		
Zarcadoolas et al 2013 [69]	x	x	x									x		x
Theme total	22	11	8	7	7	4	5	18	17	20	24	35	20	7
Category total	40						36				46			

[2] Griffin A, Skinner A, Thornhill J, Weinberger M. Patient Portals Who uses them? What features do they use? And do they reduce hospital readmissions? Appl Clin Inform. 2016;7(2):489-501. PMID: 27437056.

[3] Kruse CS, Bolton K, Freriks G. The Effect of Patient Portals on Quality Outcomes and Its Implications to Meaningful Use: A Systematic Review. J Med Internet Res. 2015;17(2):70-7. PMID: 25669240.

[4] Aljabri D, Dumitrascu A, Burton MC, White L, Khan M, Xirasagar S, Horner R, Naessens J. Patient portal adoption and use by hospitalized cancer patients: a retrospective study of its impact on adverse events, utilization, and patient satisfaction. BMC Med Inform Decis Mak. 2018;18(1):12. PMID: 30053809.

[5] Hefner JL, Sieck CJ, Walker DM, Huerta TR, McAlearney AS. System-Wide Inpatient Portal Implementation: Survey of Health Care Team Perceptions. JMIR Med Inf. 2017;5(3):e31. PMID: 28912115

[6] Kelly MM, Coller RJ, Hoonakker PLT. Inpatient Portals for Hospitalized Patients and Caregivers: A Systematic Review. J Hosp Med. 2018;13(6):405-12. PMID: 29261819.

[7] Otte-Trojel T, de Bont A, Rundall TG, van de Klundert J. What do we know about developing patient portals? a systematic literature review. J Am Med Inf Assoc. 2016;23(E1):E162-E8. PMID: 26335985.

[9] Ammenwerth E, Schnell-Inderst P, Hoerbst A. The Impact of Electronic Patient Portals on Patient Care: A Systematic Review of Controlled Trials. J Med Internet Res. 2012;14(6):325-37. PMID: 23183044.

[16] Irizarry T, Dabbs AD, Curran CR. Patient Portals and Patient Engagement: A State of the Science Review. J Med Internet Res. 2015;17(6). PMID: 26104044.

[17] O'Leary KJ, Lohman ME, Culver E, Killarney A, Smith GR, Jr., Liebovitz DM. The effect of tablet computers with a mobile patient portal application on hospitalized patients' knowledge and activation. J Am Med Inf Assoc. 2016;23(1):159-65. PMID: 26078412.

[18] Prey JE, Woollen J, Wilcox L, Sackeim AD, Hripcsak G, Bakken S, Restaino S, Feiner S, Vawdrey DK. Patient engagement in the inpatient setting: a systematic review. J Am Med Inf Assoc. 2014;21(4):742-50. PMID: 24272163.

[19] Vawdrey DK, Wilcox LG, Collins SA, Bakken S, Feiner S, Boyer A, Restaino SW. A tablet computer application for patients to participate in their hospital care. AMIA Annual Symposium proceedings AMIA Symposium. 2011;2011:1428-35. PubMed PMID: 22195206.
[20] Dumitrascu AG, Burton MC, Dawson NL, Thomas CS, Nordan LM, Greig HE, Aljabri DI, Naessens JM. Patient portal use and hospital outcomes. J Am Med Inf Assoc. 2018;25(4):447-53. PMID: 29300961.
[24] Adler-Milstein J, Holmgren AJ, Kralovec P, Worzala C, Searcy T, Patel V. Electronic health record adoption in US hospitals: the emergence of a digital "advanced use" divide. J Am Med Inf Assoc. 2017;24(6):1142-8. PMID: 29016973.
[25] Arnold CW, McNamara M, El-Saden S, Chen S, Taira RK, Bui AAT. Imaging informatics for consumer health: towards a radiology patient portal. J Am Med Inf Assoc. 2013;20(6):1028-36. PMID: 23739614.
[27] Borbolla D, Del Fiol G, Taliercio V, Otero C, Campos F, Martinez M, Luna D, Quiros F. Integrating personalized health information from MedlinePlus in a patient portal. Studies in health technology and informatics. 2014;205:348-52. PubMed PMID: 25160204.
[28] Caine K, Kohn S, Lawrence C, Hanania R, Meslin EM, Tierney WM. Designing a Patient-Centered User Interface for Access Decisions about EHR Data: Implications from Patient Interviews. Journal of General Internal Medicine. 2015;30:S7-S16. PMID: 25480719.
[29] Essen A, Scandurra I, Gerrits R, Humphrey G, Johansen MA, Kiergegaard P, Koskinen J, Liaw ST, Odeh S, Ross P, Ancker JS. Patient access to electronic health records: Differences across ten countries. Health Policy Technol. 2018;7(1):44-56. DOI: 10.1016/j.hlpt.2017.11.003.
[30] Giardina TD, Menon S, Parrish DE, Sittig DF, Singh H. Patient access to medical records and healthcare outcomes: a systematic review. J Am Med Inf Assoc. 2014;21(4):737-41. PMID: 24154835.
[33] Grossman LV, Choi SW, Collins S, Dykes PC, O'Leary KJ, Rizer M, Strong P, Yen PY, Vawdrey DK. Implementation of acute care patient portals: recommendations on utility and use from six early adopters. J Am Med Inf Assoc. 2018;25(4):370-9. PMID: 29040634.
[34] Hazara AM, Bhandari S. Barriers to patient participation in a self-management and education website Renal PatientView: A questionnaire-based study of inactive users. Int J Med Inform. 2016;87:10-4. PMID: 26806707.
[35] Hefner JL, Sieck CJ, McAlearney AS. Training to Optimize Collaborative Use of an Inpatient Portal. Appl Clin Inform. 2018;9(3):558-64. PMID: 30045386.
[36] Heyworth L, Paquin AM, Clark J, Kamenker V, Stewart M, Martin T, Simon SR. Engaging patients in medication reconciliation via a patient portal following hospital discharge. J Am Med Inf Assoc. 2014;21(E1):E157-E62. PMID: 24036155.
[39] Kelly MM, Hoonakker PLT, Dean SM. Using an inpatient portal to engage families in pediatric hospital care. J Am Med Inf Assoc. 2017a;24(1):153-61. PMID: 27301746.
[40] Kelly MM, Dean SM, Carayon P, Wetterneck TB, Hoonakker PLT. Healthcare Team Perceptions of a Portal for Parents of Hospitalized Children Before and After Implementation. Appl Clin Inform. 2017b;8(1):265-78. PMID: 28293685.
[42] Lee J, Kim JGB, Jin M, Ahn K, Kim B, Kim S, Kim J. Beneficial Effects of Two Types of Personal Health Record Services Connected With Electronic Medical Records Within the Hospital Setting. Comput Inform Nurs. 2017;35(11):574-81. PMID: 28548973.

[43] Mikles SP, Mielenz TJ. Characteristics of electronic patient-provider messaging system utilisation in an urban health care organisation. Journal of innovation in health informatics. 2014;22(1):214-21. PMID: 25924551.
[44] Nazi KM, Hogan TP, McInnes DK, Woods SS, Graham G. Evaluating Patient Access to Electronic Health Records Results From a Survey of Veterans. Medical Care. 2013;51(3):S52-S6. PMID: 23407012.
[45] Osborn CY, Mayberry LS, Mulvaney SA, Hess R. Patient Web Portals to Improve Diabetes Outcomes: A Systematic Review. Current Diabetes Reports. 2010;10(6):422-35. PMID: 20890688.
[47] Pillemer F, Price RA, Paone S, Martich GD, Albert S, Haidari L, Updike G, Rudin R, Liu D, Mehrotra A. Direct Release of Test Results to Patients Increases Patient Engagement and Utilization of Care. Plos One. 2016;11(6). PMID: 27337092.
[48] Powell KR. Patient-Perceived Facilitators of and Barriers to Electronic Portal Use: A Systematic Review. Comput Inform Nurs. 2017;35(11):565-73. PMID: 28723832.
[50] Ralston JD, Silverberg MJ, Grothaus L, Leyden WA, Ross T, Stewart C, Carzasty S, Horberg M, Catz SL. Use of Web-Based Shared Medical Records Among Patients With HIV. American Journal of Managed Care. 2013;19(4):E114-E24. PMID: 23725449.
[52] Rathert C, Mittler JN, Banerjee S, McDaniel J. Patient-centered communication in the era of electronic health records: What does the evidence say? Patient Educ Couns. 2017;100(1):50-64. PMID: 27477917.
[53] Rexhepi H, Ahlfeldt R-M, Cajander A, Huvila I. Cancer patients' attitudes and experiences of online access to their electronic medical records: A qualitative study. Health Informatics Journal. 2018;24(2):115-24. PMID: 27440056.
[54] Risling T, Martinez J, Young J, Thorp-Froslie N. Evaluating Patient Empowerment in Association With eHealth Technology: Scoping Review. J Med Internet Res. 2017;19(9). PMID: 28963090.
[55] Runaas L, Bischoff E, Hoodin F, Kentor R, Ostarello L, Seyedsalehi S, Hanauer D, Choi SW. A Novel Health Informatics Tool to Improve Caregiver Activation: Findings from Pediatric BMT in a Hospital-Based Setting. Blood. 2016;128(22).
[56] Saberi P, Catz SL, Leyden WA, Stewart C, Ralston JD, Horberg MA, Grothaus L, Silverberg MJ. Antiretroviral Therapy Adherence and Use of an Electronic Shared Medical Record Among People Living with HIV. AIDS and behavior. 2015;19 Suppl 2:177-85. PMID: 25572829.
[57] Snyder CF, Blackford AL, Wolff AC, Carducci MA, Herman JM, Wu AW, PatientViewpoint Sci A. Feasibility and value of PatientViewpoint: a web system for patient-reported outcomes assessment in clinical practice. Psycho-Oncology. 2013;22(4):895-901. PMID: 22544513.
[59] Toscos T, Daley C, Heral L, Doshi R, Chen Y-C, Eckert GJ, Plant RL, Mirro MJ. Impact of electronic personal health record use on engagement and intermediate health outcomes among cardiac patients: a quasi-experimental study. J Am Med Inf Assoc. 2016;23(1):119-28. PMID: 26912538.
[60] van der Vaart R, Drossaert CHC, Taal E, Drossaers-Bakker KW, Vonkeman HE, van de Laar MAFJ. Impact of patient-accessible electronic medical records in rheumatology: use, satisfaction and effects on empowerment among patients. BMC Musculoskeletal Disorders. 2014;15. PMID: 24673997.

[61] van der Vaart R, Drossaert CHC, Taal E, van de Laar MAFJ. Giving rheumatology patients online home access to their electronic medical record (EMR): advantages, drawbacks and preconditions according to care providers. Rheumatology International. 2013;33(9):2405-10. PMID: 22453527.
[62] Walker DM, Menser T, Yen PY, McAlearney AS. Optimizing the User Experience: Identifying Opportunities to Improve Use of an Inpatient Portal. Appl Clin Inform. 2018a;9(1):105-13. PMID: 29444536.
[66] Woollen J, Prey J, Wilcox L, Sackeim A, Restaino S, Raza ST, Bakken S, Feiner S, Hripcsak G, Vawdrey D. Patient Experiences Using an Inpatient Personal Health Record. Appl Clin Inform. 2016;7(2):446-60. PMID: 27437053
[67] Wright A, Feblowitz J, Maloney FL, Henkin S, Ramelson H, Feltman J, Bates DW. Increasing patient engagement: patients' responses to viewing problem lists online. Appl Clin Inform. 2014;5(4):930-42. PMID: 25589908.
[68] Yen PY, Walker DM, Smith JMG, Zhou MP, Menser TL, McAlearney AS. Usability evaluation of a commercial inpatient portal. Int J Med Inform. 2018;110:10-8. PMID: 29331248.
[69] Zarcadoolas C, Vaughon WL, Czaja SJ, Levy J, Rockoff ML. Consumers' Perceptions of Patient-Accessible Electronic Medical Records. J Med Internet Res. 2013;15(8). PMID: 23978618.
